# Acne vulgaris, mental health and omega-3 fatty acids: a report of cases

**DOI:** 10.1186/1476-511X-7-36

**Published:** 2008-10-13

**Authors:** Mark G Rubin, Katherine Kim, Alan C Logan

**Affiliations:** 1 Lasky Skin Clinic, 153 Lasky Drive, Suite 1, Beverly Hills, CA 90212, USA; 2 Integrative Care Centre of Toronto, 3600 Ellesmere Road, Unit 4, Toronto, ON M1C 4Y8, Canada

## Abstract

Acne vulgaris is a common skin condition, one that is associated with significant psychological disability. The psychological impairments in acne include higher rates of depression, anxiety, anger and suicidal thoughts. Despite a paucity of clinical research, patients with skin conditions and/or mental health disorders are frequent consumers of dietary supplements. An overlap may exist between nutrients that potentially have both anti-acne and mood regulating properties; examples include omega-3 fatty acids from fish oil, chromium, zinc and selenium. Here we report on five cases of acne treated with eicosapentaenoic acid and antioxidant nutrients. Self-administration of these nutrients may have improved inflammatory acne lesions and global aspects of well-being; the observations suggest a need for controlled trials.

## Background

Acne vulgaris is a common disease in developed nations, one that has increased in frequency in the last half century, particularly among adult women [1]. While the experience of acne may not be life-threatening per se, it does carry with it significant psychological disability. Indeed the psychological sequela of acne includes higher rates of clinical depression and anxiety, anger, suicidal thoughts and even suicide itself [2-6]. Evaluations have determined that patients with acne have a more significant impairment of mental health than many other chronic medical conditions, including epilepsy and diabetes [7]. Self-esteem issues are likely the driving force behind higher rates of unemployment in acne, however there is also an existing bias whereby patients with acne are more likely to be passed over by prospective employers [8].

The precise mechanisms of the acne process are not completely understood, however it is known to be characterized by sebum overproduction, follicular hyperkeratinization, oxidative stress and inflammation. Androgens, microbes and other pathogenetic influences are also at work in the development of acne [9]. Since inflammation is one of the earliest events to occur in the acne process, the influence of inflammatory mediators and subsequent free radical generation has become a major focus of experimental and clinical research [10]. Speculation that dietary factors can influence acne has been a matter of debate for decades, and emerging research is certainly suggesting that diet may indeed be an important factor, particularly in mediating the inflammation and oxidative stress of the acne process [11-13]. Hints that omega-3 fatty acids might positively influence acne originate from older epidemiological studies which show that communities that maintain a traditional diet high in omega-3 fatty acids have low rates of acne [14]. One study in over 1000 teenagers from North Carolina found that each of the primary signs of acne – comedones, papules, pustules, acne cysts and oily skin – were significantly lower in in those consuming the greatest amounts of fish and seafood [15]. A separate investigation showed that patients actually with acne are more likely to be infrequent consumers of dietary fish and seafood [16]. More recently, investigators have outlined mechanisms whereby fish oil may be effective in reducing inflammatory acne. The inflammatory chemical leukotriene B4 (LTB4) is now know to up-regulate sebum production, and synthetic inhibition of LTB4, in the form of the drug zileuton, leads to significant improvement in acne [17,18]. Eicosapentaenoic acid (EPA) from fish oil, and gamma-linolenic acid (GLA) from borage oil, have been reported to inhibit the conversion of arachidonic acid into LTB4 to the same degree as the LTB4-inhibiting acne drug candidate zileuton [19]. Sophisticated studies have recently demonstrated that each pilosebaceous unit has the machinery in place to manufacture inflammatory chemicals, including LTB4, with the raw materials supplied from the breakdown products of dietary fats [20]. Fish oil, and EPA in particular, has a wealth of research to support its ability to inhibit LTB4 production [21], yet to date, no investigators have reported on the clinical utility of fish oil for acne.

A number of emerging studies have also shown that acne patients may be under increased local and systemic oxidative stress, and the lowered blood levels of various antioxidant and anti-inflammatory nutrients might be a reflection of the increased demand in acne [22-24]. For example, lower levels of vitamins A and E among 200 age-matched acne patients vs. controls was recently found to be associated with the severity of acne [25]. Furthermore, since the selenium-dependent glutathione peroxidase enzyme activity is low in acne patients, it has been theorized that selenium would be of value. Indeed, low levels of blood selenium have been documented in acne patients, and one study examined the effect of selenium (400 mcg) and vitamin E (20 mg) daily for 12 weeks in acne. The combination led to improvements, especially in those with low baseline glutathione peroxidase activity [26,27]. The epigallocatechin-3-gallate (EGCG) polyphenol from green tea has also been suggested to be helpful in acne due to its well documented anti-inflammatory and antioxidant activity. In addition, reports suggests EGCG may also influence hormonal aspects of acne since it is known to possess 5-α-reductase inhibiting properties when applied topically [28]. Other botanical remedies which have antioxidant and anti-inflammatory activity, such as turmeric, have a long history of use in skin disorders such as acne [29].

Various studies over the last three decades have shown that zinc levels are lower in acne patients than controls, and that oral and topical combination zinc may be of therapeutic value [30-32]. There have also been hints in the literature that insulin and blood sugar abnormalities may be involved in the promotion of acne. The therapeutic value of tolbutamide and other oral sulphonylureas in acne has resulted in some scientists referring to acne as diabetes of the skin [33]. Epidemiological studies suggest that traditional diets with a low glycemic load are protective against acne [34] and one open label trial showed that 400 mcg of chromium improves acne [35]. Clinical intervention studies show that a diet with an overall low glycemic load, more dietary fiber, more fish and seafood is helpful in acne. Results show an average 22 less acne lesions in those who adhere to such a diet [11,12].

On the one hand there are certainly hints that marine lipids, minerals and phytochemicals might have potential to reduce inflammatory acne lesions, and on the other, it is almost difficult not to notice that these are the very same nutrients that have been shown to influence mental outlook, depressive symptoms and anxiety. Omega-3 fatty acids [36], zinc [37], selenium [38], chromium [39], and even phytochemicals such as those found in green tea [40], have been shown to improve mood and decrease anxiety in various clinical and experimental studies. We, and others [41], have wondered if such dietary nutrients, or combinations thereof, might influence inflammatory acne and perhaps more importantly, the mental outlook of users with acne. Clinical studies utilizing dietary changes or specific nutrient interventions in acne have not evaluated aspects of mood or mental health as an outcome.

## Brief report

Given this background on the dietary nutrients that may mediate both the acne process and mental well-being, we decided to track users of an omega-3-based dietary supplement purported to help with acne. The supplement (*perfect skin*, Genuine Health Inc, Toronto, Canada), is readily available through internet and retail outlets, it is not, however, sold, promoted or endorsed in our clinic. Each capsule of this supplement contains 250 mg of EPA (from sardines and anchovies), 3.75 mg of zinc gluconate, 50 mcg of selenium, 50 mcg of chromium and 50 mg of EGCG from green tea extract. The total daily intake is provided in table 1.

**Table 1:** Daily intake from omega-3, poly-nutrient supplement

Eicosapentaenoic Acid	1000 mg
EGCG	200 mg
Zinc Gluconate	15 mg
Selenium	200 mcg
Chromium	200 mcg

Patients with acne and other skin conditions frequently inquire about the utility of various dietary supplements. Research shows that individuals with skin conditions and/or mental health disorders are among the highest consumers of dietary supplements [42-44]. The clinical value of such supplements remains largely uninvestigated. Over the course of one year in our high-volume clinic, we identified and followed 5 subjects with mild to moderate acne vulgaris who initiated and consistently used the omega-3-nutrient combination at the package-suggested four capsules per day, for at least two months. The capsules were self-administered by the patients, and those who agreed to be followed signed informed consent concerning our interest and evaluation of outcome as case reports. In the three males and two females (aged 18–23) who disclosed that they would be initiating the dietary supplement, we performed a total comedonal lesion and inflammatory papule count based on the acne grading system established by Allen and Smith [45]. We also had the patients complete the Arizona Integrative Outcomes Scale (AIOS). The AIOS is a validated instrument designed to detect changes in global aspects of well-being, including mental, emotional and social well-being over time. It is a broad assessment, based simply on a 10 cm visual analog scale (VAS) which is used to provide a single numerical value to these constructs of well-being [46]. During the 2-month time period of self-administration of the dietary supplement, the 5 users reported that they did not alter dietary habits or overall lifestyle, did not take any other dietary supplements, and other than a mild facial cleanser, did not initiate any oral or topical anti-acne interventions.

## Discussion

**Figure 1:**
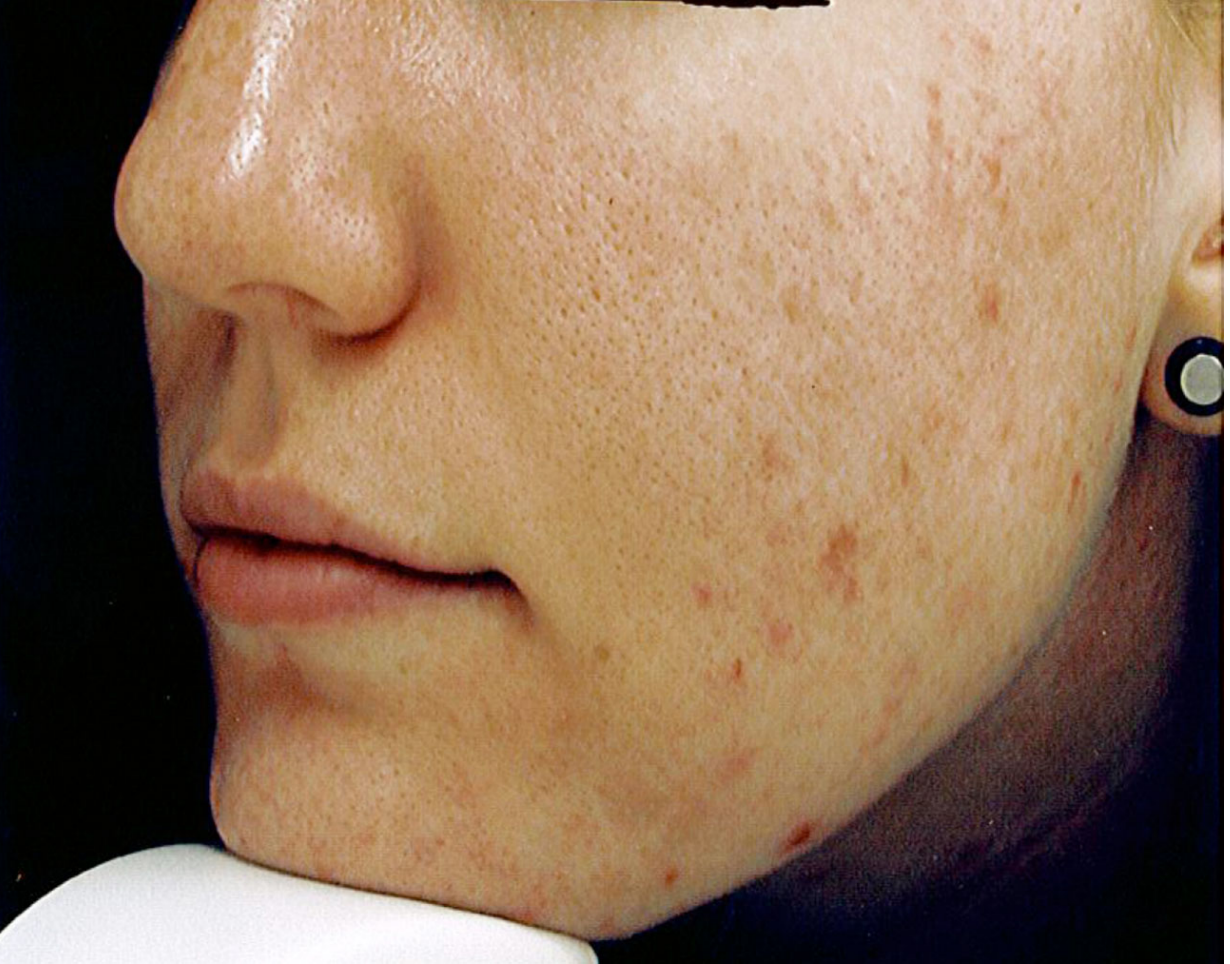
Subject photo before use of omega-3 poly-nutrient capsules

**Figure 2:**
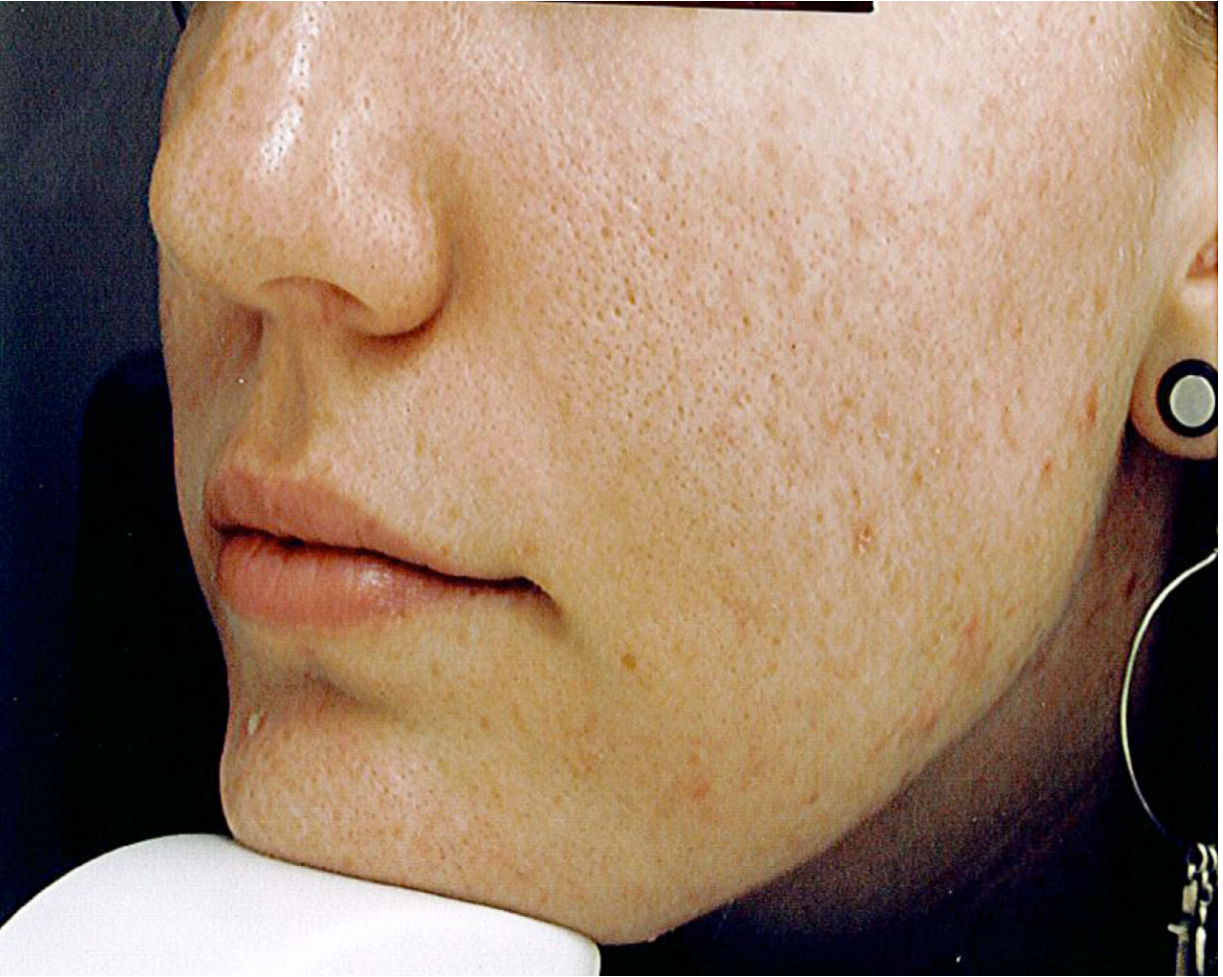
Subject photo after use of omega-3 poly-nutrient capsules

**Figure 3:**
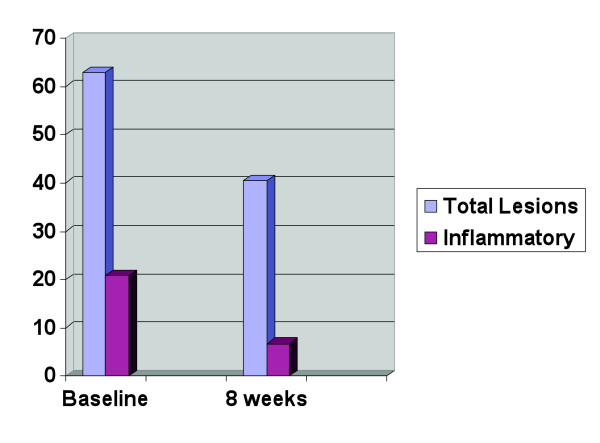
Group average total and inflammatory papule lesion counts

The self-administration of an omega-3 fish oil-based nutrient combination for two months did appear to have some influence on the acne process, and perhaps more importantly, on mental outlook. Specifically, four of the individuals had a reduction in total lesion count, with a range of 11 to 41 less lesions after 2 months. The average total lesion count among the group dropped from 62.8 to 40.4. It was in the area of inflammatory lesions where the intervention seemed to make a more significant difference. This reduction of marked inflammation and redness of lesions is apparent in the photographic evidence of figures 1, 2. Remarkably, not one subject had a worsening of inflammatory acne lesions during the two months, and all had at least some reduction in inflammatory papules. The average inflammatory lesion count at baseline was 20.8 and this decreased to 6.8 after two months. The average lesion counts are presented in the bar graphs of figure 3. One of the five subjects did have a higher overall acne lesion count after two months, there was a specific 2-fold increase in pustules and comedones, however, a 30 percent reduction in inflammatory papules was also noted.

Based on the AIOS evaluations, the omega-3-based intervention did seem to make a difference in mental outlook and global aspects of well-being over two months. We found an average 24 percent improvement in mental, emotional, and social well-being among users of the omega-3-poly-nutrient supplement. The positive changes in aspects of well-being were evenly spread among the group (range 20–27 percent) and did not appear to be associated with acne lesions, nor were they restricted to those who had the greatest reduction in acne lesions. The results are in support of a growing body of research indicating that the omega-3 fatty acid EPA can help regulate mood and depressive symptoms, even in otherwise healthy adults without clinical depression [47,48].

Our observations of these patients are only suggestive, and they hint that omega-3 fatty acids, and perhaps other nutrients in combination, may have a positive influence on inflammatory acne lesions and aspects of mental health. Obviously our decision to follow these individuals may have had a profound influence on outcome. Acne is a disease where the placebo effect has been well documented, and aspects of mental health are certainly influenced by expectations and beliefs [49]. Awareness of our interest in the outcome may have had an impact on the expectations and beliefs pertaining to the product. Although the subjects did not report changes in anti-acne interventions, dietary changes, vitamins or herbal supplement use, the effects of stress and environmental variables cannot be discounted. Emotional stress has been known to worsen acne, and this, combined with monthly hormonal fluctuations, can influence both acne and mental aspects of well-being [50].

Clearly, we can draw no conclusions from our observations in a select few cases of dietary supplement users. The results in these cases should merely serve as a motivating factor to pursue this line of research with more vigor, specifically in analyses which involve controls and determination of true statistical value. One clue that indicates the results may be more than a placebo effect is the specific, positive influence on inflammatory lesions, one that occurred in conjunction with either no significant change or even worsening of total non-inflammatory lesions. It is also true that certain acne interventions such as isotretinoin, although clinically valuable, do not improve aspects of mood, and may indeed make them worse [51]. The rationale for a double-blind, placebo-controlled clinical trial of EPA and/or anti-inflammatory nutrients in acne and mental health is apparent. Objective measurements, such as blood essential fatty acid levels, antioxidants and sebum production, will lend scientific credibility to such an investigation. The co-administration of EPA with isotretinoin should also be considered. Given the great degree of distress experienced by those with acne, any intervention that has the potential to diminish the most evident acne lesions, the inflammatory lesions, and improve mental outlook, is certainly worth investigating.

## Competing interests

MR and KK report no conflict. AL has been a compensated consultant to Genuine Health Inc, the manufacturer of the omega-3, poly-nutrient supplement used by the subjects. He has no financial interest in its sales and holds no stock or shares, nor does he possess financial holdings with Genuine Health. The product is not patented and not under active patent application.

## Authors' contributions

MR and KK performed the subject identification, clinical evaluations and coordinated the follow-ups of the subjects. AL acted as a technical expert with the properties of omega-3 fatty acids and the specific nutrients used by the subjects and he also drafted the manuscript. All authors reviewed the manuscript and agree to its content.

## Consent

All of those patients followed provided signed consent forms. The subject in figure 1 provided consent for the use of her photographic images.
